# Utilising a Behaviour Change Model to Improve Implementation of the *Activate* Injury Prevention Exercise Programme in Schoolboy Rugby Union

**DOI:** 10.3390/ijerph18115681

**Published:** 2021-05-26

**Authors:** Craig Barden, Keith A. Stokes, Carly D. McKay

**Affiliations:** 1 Department for Health, University of Bath, Bath BA2 7AY, UK; k.stokes@bath.ac.uk (K.A.S.); c.d.mckay@bath.ac.uk (C.D.M.); 2 Rugby Football Union, Twickenham TW2 7BA, UK; 3 Centre for Motivation and Health Behaviour Change, University of Bath, Bath BA2 7AY, UK

**Keywords:** youth, sport, injury, prevention, rugby, workshop, coach, activate, behaviour, implementation

## Abstract

The Health Action Process Approach (HAPA) is a behaviour change model showing promise in positively changing youth sport coaches’ injury prevention behaviours. This study incorporated the HAPA model into coach training workshops for *Activate*, an efficacious rugby injury prevention programme. Primary aims were to investigate the effect of the workshop on schoolboy rugby union coaches’ (1) perceptions towards injury risk and prevention, (2) *Activate* adoption and adherence. Secondary aims were to (3) assess the differences in post-season HAPA constructs between workshop attendees and non-attendees, (4) explore associations between HAPA constructs and *Activate* adherence. In the pre-season, all participants (*n* = 76) completed a baseline survey, with 41 coaches electing to attend a workshop. Participants completed a post-season survey assessing HAPA constructs and *Activate* adoption and adherence throughout the season. The workshop did not affect coach perceptions of injury risk and prevention. Attendees had significantly greater rates of *Activate* adoption (95% vs. 54% χ^2^ = 17.42, *p* < 0.01) and adherence (median = 2 sessions vs. ≤1 session per week; z = 3.45, *p* = 0.03) than non-attendees. At post-season, attendees had significantly greater task self-efficacy (z = −3.46, *p* < 0.05) and intention (z = −4.33, *p* < 0.05) to use *Activate*. These results support the delivery of coach workshops that utilise a behaviour change model to maximise programme implementation.

## 1. Introduction

*Activate* is a neuromuscular warm-up, efficacious at reducing injury risk in schoolboy rugby union [1,2]. In 2015, a randomised controlled trial found completing the 20-minute programme three times per week reduced match injury incidence by 72% and concussion incidence by 59% compared to a traditional warm-up [2]. The Rugby Football Union (RFU; national governing body for English rugby union) and World Rugby (international governing body for rugby union) have since been promoting *Activate* to their members. Unfortunately, efficacious interventions do not always have their desired effect once rolled out in applied settings [3,4,5]. Embedding evidence-based research into practice is extremely difficult, with behaviour influenced by contextual, social, and political factors [6,7,8]. Successful implementation of sports injury prevention programmes is complex [9], yet it fundamentally requires behaviour change in the intended end-users. 

Coaches are integral to implementing injury prevention programmes in community settings [10,11,12]. One strategy used to increase coach uptake of preventative interventions is through educational workshops. Soccer coaches attending an *11+* injury prevention programme workshop had 8 to 12% greater compliance compared to non-attendees [13,14], whilst mandatory injury prevention workshop attendance contributed to reductions in national injury rates in community rugby [15,16]. Furthermore, workshops have been associated with positive changes in coaches’ attitudes towards injury risk and prevention [17,18] and improving their self-efficacy to use an injury prevention programme [17]. Many of these studies were guided by behaviour change models to underpin, assess, and modify end-user behaviour. This approach has been historically underutilised in sports injury prevention but is gaining traction [19].

The Health Action Process Approach (HAPA) is one model that holds promise in this context [20]. Firstly, a motivational phase exists where individuals develop an intention to adopt a desired behaviour through three pre-intentional factors: risk perception (perception of threat or harm), outcome expectancy (belief that behaviour will result in a specific change), and task self-efficacy (one’s belief in their ability to change behaviour). Once an intention has been developed, a volitional phase occurs where individuals develop action (when-where-how) and coping plans (if the action plan is disrupted) to help regulate behaviour. These plans are influenced by maintenance self-efficacy (confidence to overcome potential barriers). Maintenance and recovery (confidence to continue a behaviour once suffering a setback) self-efficacy help individuals maintain their desired behaviour for the long-term once actioned. Finally, self-monitoring allows an individual to continually evaluate and regulate their behaviour [20]. These five volitional constructs are essential in minimising impulsive actions, which may result in negative outcomes [21]. 

The utility of the HAPA model has been demonstrated across a variety of health domains [22,23,24], recently including sports injury prevention [25,26]. The motivational phase of the model has been assessed in youth soccer coaches, where task self-efficacy and outcome expectancy were positively associated with greater intention to use the *11+* [26]. Volitional constructs have been partly explored in youth rugby coaches, with strong action plans leading to greater *Activate* compliance [25]. However, as both sport-specific studies were conducted as part of randomised controlled trials, there is a need to assess the utility of the HAPA model in an applied setting. 

The primary aims of this pragmatic study were to assess the effect of a pre-season coach workshop, comparing attendees (intervention) and non-attendees (control) (1) perceptions towards injury risk and prevention, and (2) coaches’ adoption (programme use; yes/no) and adherence (frequency per week) of the *Activate* injury prevention exercise programme. Secondary aims were to (3) assess differences in post-season HAPA construct scores between those who did and did not attend a workshop, and (4) provide an exploratory analysis of the relationships between HAPA model constructs and *Activate* adherence for those attending a workshop. 

## 2. Materials and Methods

### 2.1. Recruitment

Schools participating in RFU competitions (under-12 to under-19) were identified from fixtures and results on the RFU website (*n* = 289). Email addresses were sought online for school rugby coaches (including conditioning coaches and medical staff). Identified personnel were sent a recruitment email prior to the season (July–August 2018 and 2019) inviting their school to join the study. If no response was received within two weeks, they were sent a follow-up email after which it was accepted that their school did not wish to participate. The RFU publicised the study through coach correspondence and social media, encouraging coaches to contact the research team if they wished to join the study. 

For schools expressing an interest, electronic information sheets and consent forms were provided to a school gatekeeper (generally the head coach), who passed on information and links to fellow coaches. Coaches were required to provide written, informed consent prior to participation.

### 2.2. Baseline Survey

All coaches were asked to complete an online baseline survey detailing their demographics, perceptions of injury risk and prevention in rugby, and awareness of *Activate*. The survey contained 34 questions and took approximately 10 min to complete (Table S1).

Questions were adapted from studies investigating perceptions and intentions of end-users towards the *11+* [26,27,28], but were re-worded to make them specific to rugby and *Activate*. The survey consisted of single answer multiple choice questions, multiple answer multiple choice questions and the 7-point Likert scale questions. Likert scales were reversed randomly throughout the survey to prevent bias towards the left side of the scale [29]. 

### 2.3. Workshop

As part of the recruitment process, coaches were invited to attend a free injury prevention workshop, with no obligation to attend. Workshops were delivered around England in pre-season (August–September 2018 and 2019) by RFU community rugby coaches. These educators received training through RFU area training managers, ensuring delivery was consistent between workshops and deliverers. The same presentation and format were used for all workshops. 

The workshop structure and content were based on HAPA model constructs and Bandura’s theorised sources of self-efficacy [30]. Injury rates from injury surveillance studies across different age groups were initially presented (risk perception), followed by findings from the *Activate* efficacy study [2] (outcome expectancy). Coaches were shown videos of the *Activate* exercises, followed by a live demonstration from the workshop deliverer (task self-efficacy). Coaches were provided ample time outside on a pitch (45 min) to coach the exercises to a peer and assume the role of a player performing the exercises (task self-efficacy). Finally, there was a group discussion to identify potential barriers to programme use, highlighting resources to facilitate use (social support). Coaches were subsequently asked to write down action and coping plans for scenarios, which may occur as a result of the identified barriers (maintenance and recovery self-efficacy through problem solving and planning). These were subsequently shared with the group to encourage conversation.

### 2.4. Post-Workshop Survey

Immediately post-workshop, attending coaches were asked to complete a survey regarding their perceptions and intentions to use *Activate* (Table S1). Coaches not attending a workshop did not complete this survey. Surveys were administered electronically or on paper, depending on participant preference. Questions were informed by HAPA constructs and taken from a study of soccer coaches [26]. 

*Activate* was not mentioned in recruitment correspondence, or in the workshop invitation, to prevent bias in participation or in survey responses. After baseline surveys and workshops were completed, gatekeepers were sent a link to the *Activate* section of the RFU website as a resource (https://www.englandrugby.com/participation/coaching/activate, accessed on 24 May 2021). The website contains an introductory video along with demonstration videos of how to coach and perform each exercise for the programme’s various age-group versions and phases. Previously published *Activate* research was available open access [2,31]. Coaches were not instructed to use the programme as a requisite of the study.

### 2.5. Post-Season Survey

At post-season, all participants, irrespective of workshop attendance, were sent a survey (40 questions) containing the same questions used at baseline, excluding demographics. Questions detailing perceptions towards *Activate*, along with self-reported adoption and adherence during the study period, were added (Table S1).

### 2.6. Analysis

Each coach was only included in the study for one season. If a coach completed the survey in both seasons, their survey responses and workshop attendance were taken from the first season. Descriptive statistics (proportions (%), means and standard deviations (SD)) were used to summarise participant demographics. Likert scales were scored from 1 to 7, with reversed responses re-coded to ensure consistency (e.g., positive (1) to negative (7)). Ordinal data collected from Likert scale responses are presented using median and inter-quartile range (IQR). 

Participants were grouped by workshop attendance (intervention) or non-attendance (control) and compared for the primary research questions. A Wilcoxon Signed Rank test was used to assess intra-group differences between baseline and post-season perceptions towards injury risk and prevention (research question 1). Inter-group differences in coach behaviour were analysed using a Chi-Squared test for adoption (yes/no) and a Mann-Whitney-U test for adherence tertiles (≤1/2/3+ sessions per week; research question 2).

An aggregated score for each HAPA construct was calculated to allow for analysis of post-season construct scores and exploratory analysis of the HAPA model. The Likert response for each question relating to the construct were summed and divided by the maximum possible score. For example, ‘1. Strongly Agree’ + ‘3. Slightly Agree’/14 (maximum Likert response of 7 × 2 questions) = 4/14 = 0.286. Normality of aggregated construct scores were assessed by the Kolmogorov-Smirnov test (normality *p* > 0.05). Data were not normally distributed, so Mann-Whitney-U tests were used to assess differences in post-season scores between workshop attendees and non-attendees (research question 3). Cronbach’s alpha was used to assess the internal consistency of each post-season construct, regarded as unacceptable (<0.5), poor (0.5–0.6), acceptable (0.6–0.7), or good (0.7–0.9) [32]. Spearman Rank Order Correlations (ρ) were used to assess correlation between HAPA constructs for workshop attendees (research question 4). Correlation coefficients were interpreted as trivial (<0.1), small (0.1–0.3), moderate (0.3–0.5), and strong (0.5–0.7) [33]. 

A Holm-Bonferroni correction was used to counteract the risk of Type-I errors due to multiple comparisons. *p* values were adjusted with statistical significance accepted at *p* < 0.05.

## 3. Results

Baseline surveys were completed by 123 coaches; 15 were excluded due to completion after the season had started. A further 32 were excluded due to not completing the post-season survey, resulting in a final sample size of *n* = 76. Eight workshops were delivered to 41 participants over two seasons, with 35 coaches not attending a workshop. Two workshop attendees did not complete the post-workshop survey and were excluded from the exploratory analysis of the HAPA model. Participant demographics are presented by group in Table 1 (full demographic information, including those excluded from the study, is presented in Table S2). 

### 3.1. Workshop Attendance and Perceptions towards Injury Risk and Prevention

At baseline, workshop attendees and non-attendees had similar rates of *Activate* awareness (66%, 95% CI: 51–81 vs. 83%, 95% CI 71–95), previous *Activate* use (46%, 95% CI: 31–61 vs. 43%, 95% CI: 27–59), and current adoption (39%, 95% CI: 24–54 vs. 37%, 95% CI: 21–53). Both groups ‘*strongly agreed’* that rugby injuries can shorten a player’s career and cause physical problems later in life, ‘*agreeing’* that rugby injuries were preventable. No significant within-group changes in perceptions were found from baseline to post-season (Table S3). 

### 3.2. Workshop Attendance and Outcome Behaviour

Coaches attending the workshop had significantly greater *Activate* adoption during the season than non-attendees (95%, 95% CI: 88–100 vs. 54% 95% CI: 37–71; χ^2^ = 17.42, *p* < 0.01). Workshop attendees had significantly greater *Activate* adherence throughout the season (median = 2 sessions vs. ≤1 session per week, respectively; z = 3.45, *p* = 0.03). 

### 3.3. Workshop Attendance and Post-Season HAPA Constructs

Internal consistency for survey items capturing the HAPA constructs are presented in Table 2. Risk perception had an unacceptable level of internal consistency (α = 0.444) and was removed from further analysis of post-season construct scores. Workshop attendees had significantly greater task self-efficacy and intention to use *Activate* than non-attendees (Table 2). No other significant differences were found. 

### 3.4. Exploratory Analysis of HAPA Constructs and Activate Adherence

**Figure 1:**
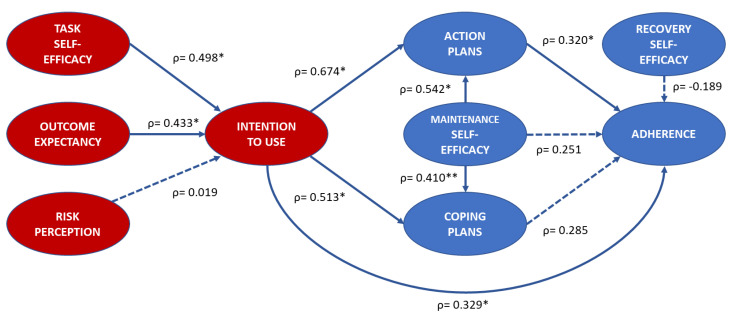
Spearman Rank Order Correlation (ρ) for post-season HAPA construct scores. Solid line signifies * *p* < 0.05.

Correlations between pre-season HAPA constructs and *Activate* adherence for workshop attendees are presented in Figure 1. Internal consistency of constructs was not assessed for this research question due to its exploratory nature and low sample size (*n* = 39). Task self-efficacy and outcome expectancy had moderate correlations with intention to use *Activate*, whilst risk perception had a trivial correlation. Intention had strong correlations with both planning constructs and *Activate* adherence. There was a strong correlation between maintenance self-efficacy and action planning, which subsequently had a moderate correlation with adherence. All remaining proximal constructs had a small correlation with adherence. 

## 4. Discussion

This study describes the effect of a pre-season workshop on coach perceptions towards rugby injury risk, prevention, and uptake of the *Activate* injury prevention exercise programme. The workshop did not change coaches’ perceptions of injury risk and prevention from baseline to post-season; however, workshop attendees had significantly greater *Activate* adoption and adherence during the study season. This supports utilising a coach workshop as a behaviour change strategy to improve *Activate* implementation in school rugby. 

Coaches from both groups were generally aware of *Activate* at baseline. This is positive, given *Activate* is in relative infancy compared to more established programmes, which have been blighted by poor coach awareness [34,35]. Still, less than half of the coaches reported previously or currently using *Activate*. Improving coach awareness is critical for programme implementation, especially as they often act as delivery-agents in community settings [10,12]. Yet, awareness alone is not enough for coaches to adopt such interventions [18,36] and evaluation of psychological and contextual factors is needed to further understand influences on their behaviour [7,37]. 

Coaches in both groups demonstrated awareness that rugby injuries can have a detrimental effect on a player’s career [38], quality of life [38,39], and team performance [40]. Coaches displayed knowledge that completing a rugby-specific warm-up can reduce injury risk [2,31]. No significant change in perceptions towards injury risk and prevention were found in either group from baseline to post-season. The lack of change in non-attendees’ perceptions is unsurprising as no intervention was administered to these participants. Coaches who attended a workshop demonstrated good baseline knowledge of injury risk and prevention and it may be significant changes were unobtainable due to a ceiling effect. It could also be that the workshop is an inadequate strategy to change attendee perceptions. However, workshop attendees had significantly greater *Activate* adoption and adherence during the study season. This suggests that the positive rates of *Activate* uptake by workshop attendees is not associated with perception changes. Instead, other behavioural determinants appear to be salient in this context. For instance, a large proportion of workshop time was dedicated towards improving coach self-efficacy, utilising Bandura’s [30] sources of self-efficacy: mastery experience, verbal persuasion, vicarious experience, and physiological states. At post-season, workshop attendees had significantly greater task self-efficacy and intention to use *Activate*. Moreover, exploratory analysis of the motivational phase of the HAPA model found task self-efficacy and outcome expectancy were strongly correlated with intention to use *Activate*. This finding is consistent with the literature [25,26]. Although it is unclear whether the workshop directly improved self-efficacy, a recent study of soccer coaches found that workshop attendance significantly improved task self-efficacy towards using the *11+* [17]. It is also likely that intervention uptake further enhances task self-efficacy for coaches implementing the programme over time. Therefore, self-efficacy may be a more potent target for coach behaviour change during injury prevention programme implementation.

The findings also reinforce the need to bolster other HAPA constructs as part of our implementation efforts. Action planning appears to be strongly associated with intention, maintenance self-efficacy, and *Activate* adherence. Only one previous study has investigated the volitional phase of HAPA during sports injury prevention programme use. In a sample of school rugby coaches, those with stronger intentions (PR = 1.49, 90% CI: 1.11–2.00) and action plans (PR = 1.33, 90% CI: 0.93–1.89) had greater *Activate* compliance [25]. This present study found that intention had a moderate correlation with adherence. The postulated relationship between intention and behaviour has been questioned, termed the intention-behaviour gap [18], which may explain why individuals with good intentions fail to uptake health-related interventions [4]. However, these results do not negate the value of the volitional phase of the HAPA model. Sports injury prevention programmes require adoption, adherence, and maintenance over prolonged periods of time [41,42]. Proximal HAPA constructs are postulated to influence these long-term outcomes, particularly when there are barriers to use or a behaviour relapses [20,21]. The associations found between volitional constructs supports the applicability of this part of the model in a school rugby context. Programme adherence requires an end-user to regulate their behaviour over a period of time. Maintenance self-efficacy was strongly associated with action planning, which was strongly associated with adherence. Some behaviour change models used in the sports injury prevention field, such as the Health Belief Model and the Theory of Planned Behaviour [43], fail to evaluate mediators between intention and behaviour. Whilst favourable results have been reported using these behaviour change models, the results from this present study suggest that improving post-intentional cognitions, such as maintenance self-efficacy and action planning, may enhance outcome behaviour. However, the results of this exploratory analysis should not be overstated with limitations around sample size and internal consistency of some constructs.

Overall, these findings promote utilising coach workshops as a strategy to improve injury prevention implementation. Targeting rugby coaches through workshops is further supported by evidence that they impart intervention awareness [44] and knowledge onto their players, resulting in positive changes towards safe tackling, scrummaging [45,46], and rucking [15]. The Australian [47], New Zealand [15], and South African [45] rugby unions have made injury prevention workshops compulsory for coaches, and these have been associated with significant reductions in nationwide injury rates [15,16]. Making attendance at an *Activate* workshop compulsory may lead to greater reductions in injury risk in English school rugby.

### Limitations

The school recruitment database was extensive (*n* = 289) but only coaches from 25 different schools participated, limiting the external validity and generalisability of the results. Self-selection bias may have occurred in those electing to attend a workshop. To minimise this bias, *Activate* was not mentioned in any recruitment or workshop correspondence. Individuals were not instructed to use the intervention to participate in the study. Given a third of workshop attendees had not previously heard of *Activate* at baseline, it is unlikely these coaches would have used the programme if they had not attended a workshop.

Likert scale surveys are prone to several biases. Participants are more likely to avoid extreme ends of the scale (central tendency bias) [48], leaving researchers unable to differentiate between individuals. Whilst a 7-point Likert scale was used to minimise this bias, it is possible that it was related to the lack of within-group change in perceptions from baseline to post-season. Evidence suggests that respondents favour the left-side of the scales [29]. Positively worded questions, with favourable responses on the left-side of the scale, could lead to inflated levels of agreement. This bias was negated with response scales randomly reversed throughout the survey.

Participants were grouped by workshop attendance. Non-attendees were not required to complete a post-workshop survey as no control intervention was used for this pragmatic study. It was hypothesised that baseline *Activate* awareness would be low and thus it was not deemed appropriate to ask non-attendees questions detailing their perceptions or intentions to use *Activate* at this timepoint. Differences in post-season construct scores were compared between groups (research question 3). Whilst this method fails to assess for change from post-workshop to post-season, it is unlikely their baseline construct scores would have been significantly different given participants from both groups appear homogeneous.

Participants were asked to report their *Activate* use in the post-season survey, with all surveys completed within six months of the season completion. Given this relatively short period of time, it is not anticipated recall bias [49] would have affected the results of this study. However, there is a risk of social desirability bias in questions relating to *Activate* use. The reliability of coach reported *Activate* adoption and adherence could not be assessed, as it was not possible to observe practice in this context. This bias may have been greater in those attending an *Activate* workshop as the research team organised the workshops and collected survey responses. 

The study surveys were an amalgamation of those used in soccer [14,26,27] and rugby studies [2,31]. They have only been assessed for face and content validity. Whilst this study supports the use of the HAPA model in this context, the construct validity of the surveys needs to be assessed before it can be fully advocated. Intention and recovery self-efficacy were assessed using a single question. These constructs may be prone to greater response bias than constructs which were evaluated using two to three questions. The internal consistency of these constructs could not be assessed due to having only one question, furthering the need to assess the construct validity of these questions. The internal consistency of risk perception at post-season was deemed unacceptable, with outcome expectancy rated poor. This may be due to small sample size, the low number of items forming this construct or poor inter-relatedness between items [50]. 

## 5. Conclusions

Workshops are an effective implementation strategy to improve coaches’ adoption and adherence of the *Activate* injury prevention exercise programme. The workshop, strongly influenced by the HAPA model, did not change coach perceptions towards injury risk or perception. Instead, the change in behaviour is likely associated with enhancement in psychological variables such as task self-efficacy and intention. These findings support integrating behaviour change theories in the development of implementation strategies. 

## Figures and Tables

**Table 1:** Participant demographics by workshop attendance.

Information/Question	Response	Workshop Attendance	
		No *n* (%)	Yes *n* (%)
**School type**	Independent (private)	26 (74%)	36 (88%)
	State (government funded)	9 (26%)	5 (12%)
**Age of participant**	Mean Age (SD)	36.8 (±10.8)	38.6 (±10.3)
**What is your role?**	Director of Sport/Rugby	5 (14%)	2 (5%)
	Head Coach	15 (43%)	13 (32%)
	Assistant Coach	7 (20%)	19 (46%)
	Team manager	3 (9%)	5 (12%)
	Strength and conditioner	2 (6%)	0 (0%)
	Medical practitioner	3 (9%)	2 (5%)
**If coaching, what age groups do you coach?**			
	Under-12/13	2 (7%)	2 (6%)
	Under-14/15	5 (19%)	11 (32%)
	Under-16	2 (7%)	3 (9%)
	Under-17/18/19	8 (30%)	11 (32%)
	Various	10 (37%)	7 (21%)
**If coaching, how many years coaching experience do you have?**			
	Less than 2 years	3 (11%)	3 (9%)
	2–3 years	1 (4%)	4 (12%)
	4–5 years	3 (11%)	3 (9%)
	6+ years	20 (74%)	24 (70%)
**What is the highest coaching qualification you hold?**			
	RFU Level 1	2 (7%)	8 (23%)
	RFU Level 2	11 (41%)	12 (35%)
	RFU Level 3	7 (26%)	7 (21%)
	RFU Level 4	3 (11%)	1 (3%)
	None	3 (11%)	5 (15%)
	Unknown	1 (4%)	1 (3%)
**Have you ever used a specific programme to reduce injury risk amongst your players?**			
	No	22 (63%)	24 (59%)
	Yes	13 (37%)	17 (41%)

**Table 2:** Post-season construct score with 95% confidence intervals.

Construct	Questions	Workshop Attendance		Z Score	Cronbach’s Alpha (α)
		No	Yes		
Outcome Expectancy	2	0.32 (0.27–0.37)	0.27 (0.24–0.30)	−1.49	0.585
Task Self-efficacy	2	0.31 (0.27–0.35)	0.22 (0.19–0.24)	−3.46 *	0.625
Intention	1	0.47 (0.40–0.54)	0.29 (0.24–0.34)	−4.33 *	-
Action Planning	2	0.49 (0.44–0.55)	0.39 (0.34–0.43)	−2.61	0.716
Coping Plans	2	0.53 (0.48–0.58)	0.42 (0.37–0.47)	−3.21	0.810
Maintenance Self-efficacy	3	0.48 (0.42–0.54)	0.39 (0.34–0.43)	−2.28	0.733
Recovery Self-efficacy	1	0.44 (0.37–0.50)	0.36 (0.32–0.40)	−1.75	-

Note: Cronbach’s alpha could not be calculated for constructs with a single question. Risk perception was excluded from this analysis due to poor internal consistency. * *p* < 0.05.

## Data Availability

All relevant data collected for this study is presented in the manuscript and supplementary content.

## Supplementary Materials

The following are available online at https://www.mdpi.com/article/10.3390/ijerph18115681/s1, Table S1: Survey key to show questions used at different timepoints, Table S2: Baseline participant demographics, Table S3: Coach perceptions towards rugby injury risk and prevention at baseline and post-season.
