# Supplementary material for: Utilising a Behaviour Change Model to Improve Implementation of the Activate Injury Prevention Exercise Programme in Schoolboy Rugby Union
Source: Int J Environ Res Public Health. 2021 May 26;18(11):5681. doi: 10.3390/ijerph18115681 (PMC8199066; doi:10.3390/ijerph18115681)
Supplement: Supplementary file 1 [file ijerph-18-05681-s001.zip › ijerph-1231878-supplementary.pdf]

Table S1. Survey key to show questions used at different timepoints

Question/ Statement	HAPA Construct	Responses	Time Point		
			Baseline	Post-workshop	Post-season
What is your role?		**Select One:**<br>Director of Sport<br>Head coach<br>Assistant coach<br>Team manager<br>Conditioning Coach<br>Medical practitioner<br>Other	A		
If coaching, how many years coaching experience do you have?		**Select One:**<br>Never<br>Less than 2 years<br>2-3 years<br>4-5 years<br>6 years+	A		
- What is the highest level you have coached?		**Select One:**<br>School/club<br>Regional academy<br>County/constituent body<br>Divisional<br>Professional<br>International	A		
- What is the highest coaching qualification you hold?		**Select One:**<br>RFU Level 1<br>RFU Level 2<br>RFU Level 3<br>RFU Level 4<br>Other	A		
- When was this coaching qualification attained (please tick 1 answer)?		**Select One:**<br>Less than 2 years<br>2-3 years<br>4-5 years<br>More than 5 years ago	A		
- What age group do you coach/play in?		**Select One:**<br>Under-12<br>-<br>Under-19<br>Various	A		
When was the last time you completed an official RFU course?		**Select One:**<br>Less than 2 years ago<br>2-3 years<br>4-5 years<br>6+ years ago	A		
Have you previously played competitive rugby?		**Select One:**<br>No<br>Yes	A		
- If yes, what is the highest level you have played?		**Select One:**<br>School<br>Age group community<br>Junior academy	A		

		University<br>Adult community<br>Professional<br>International			
Do you have a current medical or first aid qualification?		**Select One:**<br>No<br>Yes	A		
Have you ever used a specific programme to reduce your players injury risk?			A		
In your opinion, ***how serious*** are the following types of Rugby injuries?<br>- Ankle sprain<br>- Knee ligament sprain<br>- Muscle strain<br>- Concussion<br>- Bone/fracture<br>- Bruise/contusion<br>- Shoulder injury		**7-point Likert Scale:**<br>1. Not serious<br>–<br>7. Very Serious			
Rugby injuries can ***shorten a player's career***		**7-point Likert Scale:**<br>1. Strongly Agree<br>–<br>7. Strongly Disagree	B		B
Rugby injuries can cause ***physical problems later in life***			B		B
Rugby injuries have a ***negative impact on team performance***			B		B
Rugby injuries have a ***negative impact on a player's quality of life***			B		B
Rugby players are at ***high risk of suffering an injury***	RP		B		B
I expect a player I coach to ***sustain a Rugby injury*** sometime during the next season	RP		B		B
It is ***possible to prevent*** some Rugby injuries	OE		C		C
Exercises which have been ***shown to prevent*** injuries should be performed by Rugby players			C		C
Exercises to prevent injuries should be ***varied and progressed*** over time			C		C
Exercises which have been ***shown to prevent*** injuries should be incorporated into Schools Rugby training			C		C
Completing a Rugby specific warm-up programme prior to every game and training session will ***reduce the risk*** of players sustaining an injury			C		C
Completing a Rugby specific warm-up programme prior to every game and training session ***will improve*** physical characteristics such as balance, agility and strength			C		C
Balance exercises can prevent Rugby injuries			C		C
Controlled jumping/landing exercises can prevent Rugby injuries			C		C
A ***warm-up jog/run*** can prevent Rugby injuries			C		C

Cutting exercises can prevent Rugby injuries			C		C
Cool-down jog/run can prevent Rugby injuries			C		C
Resisted shoulder exercises can prevent ***shoulder injuries*** in Rugby			C		C
Neck muscle strengthening can prevent ***concussion*** in Rugby			C		C
I would like my team to complete a Rugby specific warm-up programme prior to every game and training session next season			C		C
Who is responsible for Rugby injury prevention?<br>- Head coach<br>- Assistant coach<br>- Team manager<br>- Strength and conditioning/ fitness coach<br>- School medical practitioner<br>- Player<br>- Parent/guardian		**Tick all that apply**			
Had you ***heard of 'Activate'*** before taking part in this survey?		**Select One:**<br>Yes<br>No<br>Unsure	D		
- If yes, where did you heard about it?<br>- RFU Website<br>- Community Rugby Coaches<br>- Peers<br>- Social media		**Tick all that apply**			
Have you ***ever used*** 'Activate'?		**Select One:**<br>Yes<br>No<br>Unsure	D		
Does your team ***currently use*** 'Activate'?			D		E
Does 'Activate' need to be ***improved for use*** in your team?					
Should your school ***develop its own version*** of 'Activate'?					
'Activate' can ***prevent*** Rugby injuries in your team	**OE**	**7-point Likert Scale:**<br>1. Strongly Agree<br>–<br>7. Strongly Disagree		E	E
'Activate' is ***Rugby specific***					
'Activate' is ***too long***					
'Activate' contains adequate ***variation and progression*** for our team					
'Activate' could be ***maintained*** over multiple seasons by our team					
In your opinion, what happened to your players' ***overall risk of injury*** as they		**7-point Likert Scale:**<br>1. Extremely Increase			

completed 'Activate' in training or prior to match play playing this season?		-<br>7. Extremely Decreased			
How confident are you that ***you understand 'Activate'*** well enough to use it with the School team(s) you are coaching for the upcoming season?	**TSE**	**7-point Likert Scale:**<br>1. Extremely Confident<br>-<br>7. Extremely Unconfident		E	E
How confident are you that ***you have the ability to use 'Activate'*** with the School team(s) you are coaching for the upcoming season?	TSE			E	E
What ***resources were available*** to help you complete 'Activate' with your team at every game and training session during the upcoming season?		Free Text Box			
What ***barriers prevented or impeded you*** from the completing 'Activate' with your team at every game and training session during the upcoming season?		Free Text Box			
If there was ***limited space*** at your practice or game venue, how confident were you that your team could complete 'Activate'?	**MSE**	**7-point Likert Scale:**<br>1. Extremely Confident<br>-<br>7. Extremely Unconfident		E	E
If the players on your team ***did not enjoy*** performing 'Activate', how confident were you that you could still have them complete it at every game and training session?	MSE			E	E
If you were to start the season by completing 'Activate' with your team at every game and training session, how confident are you that you could continue to do so for the ***entire season***?	MSE			E	E
If you ***missed completing the programme*** at a game or training, how confident were you that you could start completing it again the next session?	RSE			E	E
On average, how ***many times a week*** did your team complete 'Activate' ***during this season***?		**Select One:**<br>Never<br><1 x per week<br>1 x per week<br>2 x per week<br>3 x per week			E
On average, ***how long did it take*** your team to complete 'Activate' ***during training***?		**Select One:**<br>We didn't<br>5-10 minutes<br>10-15 minutes<br>15-20 minutes<br>>20 minutes			
On average, ***how long did it take*** your team to complete 'Activate' ***before matches***?					
What are some of the things you ***did like*** about doing 'Activate' with your team before training or matches?		**Tick all that apply**:<br>Various prefilled responses			
What were some of the things you ***did not*** like about doing 'Activate' with your team before training or matches (*tick as many as appropriate*).					

What ***changes would you recommend*** to 'Activate' in terms of content or delivery structure					
Do you intend to make ***injury prevention a priority*** for your team in the upcoming season?		**7-point Likert Scale:** 1. Extremely Likely - 7. Extremely Unlikely			
Do you ***intend to complete*** 'Activate' with your team at every game and training session in the upcoming season?	INT			E	E
Do you have concrete plans on how to ***teach*** 'Activate' to the players on your School team?	AP	**7-point Likert Scale:** 1. Strongly Agree – 7. Strongly Disagree		E	E
Do you have concrete plans on ***when*** to have your team complete 'Activate'?	AP			E	E
Do you have concrete plans on how to ***deal with challenges*** in order to stick to your intentions of completing 'Activate'?	CP			E	E
Do you have concrete plans on how to ***encourage your team*** to perform 'Activate' to the best of their ability?	CP			E	E

Abbreviation Key:

TSE = task self-efficacy
OE = outcome expectancy
RP = risk perception
INT = intention
AP = action plans
CP = coping plans
MSE = maintenance self-efficacy
RSE = recovery self-efficacy
ADH = Adherence

Colour Key:

- Green signifies questions asked for this present study, labelled by section…
    - A = demographic
    - B = perceptions towards injury risk in rugby
    - C = perceptions towards injury prevention in rugby
    - D = baseline awareness and use of Activate
    - E = psychological variables/perceptions and Activate uptake
- Amber signifies questions asked for a sub-study, which were not included or analysed as part of this study.

Table S2. Baseline participant demographics

Information/Question	Response	Workshop Attendance No n (%)	Workshop Attendance Yes n (%)	Excluded from study
**School type**	Independent (private)	26 (74%)	36 (88%)	39 (83%)
	State (government funded)	9 (26%)	5 (12%)	8 (17%)
**Age of participant**	Mean Age (SD)	36.8 (±10.8)	38.6 (±10.3)	36.6 (±10.5)
**What is your role?**	Director of Sport/Rugby	5 (14%)	2 (5%)	2 (4%)
	Head Coach	15 (43%)	13 (32%)	20 (43%)
	Assistant Coach	7 (20%)	19 (46%)	14 (30%)
	Team manager	3 (9%)	5 (12%)	7 (15%)
	Strength and conditioner	2 (6%)	0 (0%)	0 (0%)
	Medical practitioner	3 (9%)	2 (5%)	4 (8%)
**If coaching, what age groups do you coach?**				
	Under-12/13	2 (7%)	2 (6%)	4 (11%)
	Under-14/15	5 (19%)	11 (32%)	11 (31%)
	Under-16	2 (7%)	3 (9%)	2 (6%)
	Under-17/18/19	8 (30%)	11 (32%)	6 (17%)
	Various	10 (37%)	7 (21%)	13 (36%)
**If coaching, how many years coaching experience do you have?**				
	Less than 2 years	3 (11%)	3 (9%)	6 (17%)
	2-3 years	1 (4%)	4 (12%)	4 (11%)
	4-5 years	3 (11%)	3 (9%)	3 (8%)
	6+ years	20 (74%)	24 (70%)	22 (61%)
	Unknown	0 (0%)	0 (0%)	1 (3%)
**If coaching, what is the highest level you have coached?**				
	School/Club	12 (44%)	19 (56%)	20 (57%)
	Regional Junior Academy	5 (19%)	4 (12%)	3 (9%)
	County/ constituent body	4 (15%)	6 (17%)	4 (11%)
	Divisional	3 (11%)	3 (9%)	3 (9%)
	Professional	1 (4%)	1 (3%)	1 (3%)
	International	2 (7%)	1 (3%)	4 (11%)
**What is the highest coaching qualification you hold?**				
	RFU Level 1	2 (8%)	8 (24%)	4 (11%)
	RFU Level 2	11 (42%)	12 (36%)	17 (47%)
	RFU Level 3	7 (27%)	7 (21%)	5 (14%)
	RFU Level 4	3 (12%)	1 (3%)	2 (6%)
	None	3 (12%)	5 (15%)	2 (6%)
	Unknown	1 (4%)	1 (3%)	6 (17%)
**When did you obtain this qualification?**				
	Less than 2 year ago	5 (22%)	9 (32%)	6 (23%)
	2-3 years ago	3 (13%)	3 (11%)	4 (15%)
	4-5 years ago	7 (30%)	5 (18%)	6 (23%)
	More than 5 years ago	8 (35%)	11 (39%)	10 (39%)
**When did you last complete an official RFU course?**				
	Never	2 (6%)	4 (10%)	7 (15%)
	Less than 2 years ago	25 (71%)	28 (70%)	29 (62%)
	2-3 years ago	4 (11%)	2 (5%)	5 (11%)
	4-5 years ago	1 (3%)	3 (8%)	4 (8%)
	6+ years ago	3 (9%)	3 (8%)	2 (4%)
**Have you previously played competitive rugby?**				

	No	0 (0%)	5 (12%)	9 (19%)
	Yes	35 (100%)	36 (88%)	38 (81%)
**If yes, what is the highest level you have played?**				
	School	2 (6%)	6 (17%)	6 (16%)
	Age group community club	1 (3%)	1 (3%)	2 (5%)
	Junior academy Rugby	1 (3%)	1 (3%)	1 (3%)
	University	6 (17%)	4 (11%)	5 (13%)
	Adult community club	17 (49%)	19 (53%)	17 (45%)
	Professional	5 (14%)	3 (8%)	5 (13%)
	International	3 (9%)	2 (6%)	2 (5%)
**Do you have a current medical or first aid qualification?**				
	No	6 (28%)	15 (37%)	15 (32%)
	Yes	29 (83%)	26 (63%)	32 (68%)
**Have you ever used a specific programme to reduce injury risk amongst your players?**				
	No	22 (63%)	24 (59%)	27 (57%)
	Yes	13 (37%)	17 (41%)	20 (43%)

Table S3. Coach perceptions towards rugby injury risk and prevention at baseline and post-season.

Statement	Workshop Attendance	Baseline		Post-season	
		Median	IQR	Median	IQR
Rugby injuries can shorten a player's career	No	1	1-2	2	1-2
	Yes	1	1-1.5	1	1-2
Rugby injuries can cause physical problems later in life	No	1	1-2	2	1-2
	Yes	1	1-2	1	1-2
Rugby injuries have a negative impact on team performance	No	2	2-5	3	2-3
	Yes	2	2-3	2	2-4
Rugby injuries have a negative impact on a player's quality of life	No	2	2-3	2	2-3
	Yes	2	2-3	2	2-3
Rugby players are at high risk of suffering an injury	No	3	2-4	3	2-5
	Yes	2	2-3	2	2-5
I expect a player I coach to sustain a injury sometime during the next season	No	2	2-3	2	2-4
	Yes	3	2-4	3	2-4
It is possible to prevent some rugby injuries	No	2	1-2	2	1-2
	Yes	2	1-2	2	1-2
**Exercises which have been shown to prevent injuries….**					
…should be performed	No	2	1-2	1	1-2
	Yes	2	1-2	1	1-2
…varied and progressed	No	1	1-2	1	1-2
	Yes	2	1-2	2	1-2
…incorporated in schools training	No	2	1-2	2	1-4
	Yes	2	1-2	1	1-2
**Completing a rugby specific warm-up prior to every game and training session will...**					
...reduce the risk of players sustaining an injury	No	2	1-2	2	2-2
	Yes	2	1-2	2	1-2
...improve physical characteristics such as balance, agility and strength	No	2	2-3	2	2-3
	Yes	2	1-2	2	1-2
Balance exercises can prevent Rugby injuries	No	2	2-3	2	2-3
	Yes	2	2-2	2	1-2
Controlled jumping/landing exercises can prevent Rugby injuries	No	2	2-3	2	1-3
	Yes	2	2-2	2	1-2
A warm-up jog/run can prevent Rugby injuries	No	3	2-4	3	2-4
	Yes	3	2-4	3	2-3
Cutting exercises can prevent Rugby injuries	No	3	2-4	2	2-3
	Yes	4	2-4	2	2-4
Cool-down jog/run can prevent Rugby injuries	No	3	2-4	3	2-5
	Yes	3	2-3	2	2-3
Resisted shoulder exercises can prevent shoulder injuries in Rugby	No	2	2-3	2	2-3
	Yes	2	2-2.5	2	2-2
Neck muscle strengthening can prevent concussion in Rugby	No	3	2-4	3	2-4
	Yes	2	2-4	2	2-3
	No	2	1-3	2	1-2

I would like my team to complete a Rugby specific warm-up programme prior to every game and training session next season	Yes	2	1-2	2	1-2
